# The neuroimmune pathway of high-altitude adaptation: influence of erythrocytes on attention networks through inflammation and the autonomic nervous system

**DOI:** 10.3389/fnins.2024.1373136

**Published:** 2024-04-04

**Authors:** Nian-Nian Wang, Si-Fang Yu, Peng Dang, Rui Su, Hao Li, Hai-Lin Ma, Ming Liu, De-Long Zhang

**Affiliations:** ^1^Key Laboratory of High Altitudes Brain Science and Environmental Acclimation, Tibet University, Lhasa, China; ^2^Key Laboratory of Brain, Cognition, and Education Sciences, Ministry of Education, Guangzhou, China; ^3^School of Psychology, Center for Studies of Psychological Application, and Guangdong Key Laboratory of Mental Health and Cognitive Science, South China Normal University, Guangzhou, China; ^4^School of Educational Sciences, Kashi University, Kashi, China

**Keywords:** attention networks, neuroimmune, heart rate variability, red blood cell count, inflammation

## Abstract

**Introduction:**

Many studies have shown that the functional adaptation of immigrants to high-altitude is closely related to oxygen transport, inflammatory response and autonomic nervous system. However, it remains unclear how human attention changes in response to hypoxia-induced neurophysiological activity during high-altitude exposure.

**Methods:**

In the present study, we analyzed the relationship between hypoxic-induced neurophysiological responses and attention networks in 116 immigrants (3,680 m) using an attention network test to simultaneously record electroencephalogram and electrocardiogram in combination with specific routine blood markers.

**Results:**

Our analysis revealed that red blood cells exert an indirect influence on the three attention networks, mediated through inflammatory processes and heart rate variability.

**Discussion:**

The present study provides experimental evidence for the role of a neuroimmune pathway in determining human attention performance at high- altitude. Our findings have implications for understanding the complex interactions between physiological and neurocognitive processes in immigrants adapting to hypoxic environments.

## 1 Introduction

The oxygen scarcity and low-pressure conditions of high-altitude regions present unique physiological challenges to the inhabitants living in these regions. Among them, erythrocytes play a crucial role in adapting to the high-altitude environment. The body improves the efficiency of oxygen carrying and delivery by compensatory increase in the number of erythrocytes to maintain the normal function of tissues (Wood and Johansen, 1972; Raberin et al., 2022; Villafuerte et al., 2022). However, in recent years, it has been shown that such compensatory changes in red blood cells (RBCs) in immigrant populations living at high-altitude may not only involve the physiological aspects of oxygen transport, but also relate to cognitive functions such as attention (Xue et al., 2022).

The brain is the most oxygen-consuming organ in the human body (Raichle and Gusnard, 2002; Reichle, 2010; Yan, 2014), and hypoxia is a stressor of human beings (Virués-Ortega et al., 2004); thus, high-altitude hypoxia exposure would induce extensive effects on brain function (Chen et al., 2017, 2021; Zhang et al., 2018a,b; Xue et al., 2022). Previous studies have demonstrated that functional adaptations to high-altitude include multidimensional physiological and neurocognitive changes, such as efficiency of oxygen usage (Yan et al., 2010; Yan, 2014; Xue et al., 2022), inflammatory response (Caris and Santos, 2019; Nguyen et al., 2021), physiological homeostasis (Beall, 2007; Frisancho, 2013), brain morphology (Virués-Ortega et al., 2004; Yan et al., 2010), and cognitive performance (Ma et al., 2015; Taylor et al., 2015; McMorris et al., 2017; Zhang et al., 2018a, 2021; Chen et al., 2021). In human cognition, attention systems have been reported to be sensitive to hypoxia (Zhang et al., 2018a,b, 2021; Chen et al., 2021; Xue et al., 2022). High-altitude exposure leads to a generalized decrease in executive attention in immigrants. Longitudinal and controlled studies comparing high- and low-altitude adults have demonstrated effects on a variety of attentional domains, including alerting, orienting, and executive control. A 2-year follow-up study showed changes in executive control efficiency in high-elevation immigrants, but no significant changes in alerting or orienting networks (Xin et al., 2017). Similarly, an assessment of a 3-year cohort of immigrants showed a decrease in executive control efficiency relative to adults at lower altitudes (Zhang et al., 2021). Another study utilizing the Stroop task found a significant reduction in migrant accuracy and delayed response times after 2 years of exposure (Chen et al., 2021). However, it remains unclear how human attention changes with hypoxia-induced neurophysiological changes during prolonged hypoxic exposure. However, it remains unclear how human attention changes in response to hypoxia-induced neurophysiological activity during long-term hypoxia exposure.

Human behavior and cognition in high-altitude environments are tightly related to oxygen transport, the inflammatory response, and the autonomic nervous system (ANS) (Goodman et al., 2011; Trimmel, 2011; Omrani et al., 2017; Pun et al., 2019; Li et al., 2020). Exposure to high-altitude could increase inflammation, such as neutrophil count and the neutrophil/lymphocyte ratio (NLR), a reliable marker of chronic low-grade inflammation (Bhat et al., 2013; Lynall et al., 2019), which is associated with decreased executive attention function (Hou et al., 2022). The inflammatory response to oxygen metabolism is regulated by the ANS (Williams et al., 2019) and the properties of RBCs. The ANS plays an important role in the neurophysiological pathway responsible for adaptively regulating inflammatory processes (Williams et al., 2019), in which HRV is usually used as an index to depict the ANS (Zhang et al., 2014). Of note, HRV is generally considered a biomarker of top-down self-regulation (Holzman and Bridgett, 2017), reflecting the capacity of regulating cognitive activity, mood, and behavior in response to changing environmental demands (Porges, 2007; Thayer and Lane, 2009). The low frequency/high frequency (LF/HF) ratio is a sensitive indicator of HRV, and an increased LF/HF ratio indicates a lower HRV (Rombold-Bruehl et al., 2019). Many studies have shown that immediate exposure to high-altitude results in increased sympathetic activity followed by a significant increase in the LF/HF ratio (Bernardi et al., 1998; Kanai et al., 2001; Chen et al., 2008; Qian et al., 2020). In addition to the HRV change from high-altitude exposure, RBCs were compensatory increased in a hypoxic environment (Wood and Johansen, 1972; Raberin et al., 2022; Villafuerte et al., 2022), and RBCs are critical players in inflammation distinct from their function in oxygen transport (Lam et al., 2021; Xu et al., 2022). Together, HRV and RBCs are closely related to oxygen transport capacity, and both are associated with inflammatory responses; the complex interactions together determine the individual's physiological homeostasis (Taylor et al., 2010). Furthermore, the changed HRV and RBCs properties were observed to be associated with poorer external attention performance (Duschek et al., 2009; Williams et al., 2016).

To examine how the hypoxia-induced neurophysiological response (oxygen transport, inflammation and ANS) connects with human attention function during the long-term exposure, the current cross-sectional study employed ANT recording EEG and ECG simultaneously, also combining specific blood routine markers to examine the three distinct attentional processes (alerting, orienting, and executive control) in 116 immigrants who had been living in Tibet (3680 m) more than 3 years. Our primary analysis examined three neural markers of attention networks, and then we used mediation analyses to explore whether physiological indicators mediated the relationship between exposure and the attention network through a series of mediation analyses. Finally, we used a path model to explore the neurophysiological pathway by which erythrocytes influence the attention network through inflammation and HRV. To exclude the possible biases due to demographic, cultural, and socioeconomic differences, we controlled for sex, BMI, and age in assessing neuropsychological/attention differences. Studying this relationship in depth, we are expected to reveal the potential effects of erythrocyte changes on cognitive functions in high-altitude environments, providing new perspectives for understanding high-altitude adaptation. This will not only help to expand our knowledge of erythrocyte physiology in high-altitude environments but may also provide important clues for the prevention and treatment of related diseases and cognitive disorders.

## 2 Methods and materials

### 2.1 Study participants

We recruited 129 Han Chinese right-handed participants who had been working or living for more than 3 years in Lhasa (3680 m) for this study. All participants were born and raised in the central plain region of China (< 1000 m), 1 subject withdrew from the physical examination due to fear of blood; 6 were excluded because of the quality of EEG data; 6 were excluded due to incomplete heart rate data. After excluding these data, statistical analysis was performed for the remaining 116 participants. None of the participants had neurological or mental illness, brain damage or drug addiction. All participants give written informed consent and are paid for their participation. The study was approved by the Ethics committee of Tibet University and follows the Declaration of Helsinki.

The average high-altitude exposure time of these participants (male: 55; female: 61) was 9.74 ± 3.61 years (range: 3-20 years), the average age was 34.55 ± 3.45 years (range: 27-41 years), and the average BMI was 22.85 ± 3.44 (range: 16.18-39.43). Their educational experiences fell into four categories: high school graduate or below, junior college, undergraduate, and postgraduate or above. Table 1 shows the demographic information of the participants in the study. And BMI, age, sex, and education levels were used as covariates in all our analyses.

**Table 1:** Demographic information for participants.

**Variables**	**Whole cohort (*N =* 116)**
Age (mean, SD)	34.55 (3.45)
Sex (Male/Female)	55/61
BMI (mean, SD)	22.85 (3.44)
**Education (%)**	
High school graduate or below	11 (9.50%)
Junior college	17 (14.70%)
Undergraduate	44 (37.90%)
Postgraduate and above	44 (37.90%)

SD, standard deviation; BMI, body mass index.

### 2.2 Experimental procedures

**Figure 1:**
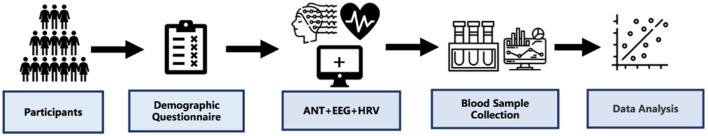
Study design. Participants completed the Demographic questionnaire, and we recorded the ANT experiment with EEG and ECG signals continuously. Then, the participants were fasted overnight, and their blood samples were collected at the next day.

At the beginning of the experiments, participants completed the demographic questionnaire, and the ANT experiment with EEG and ECG signals were recorded continuously with an ANT Neuro 64-electrode system and BioHarness Physiology Monitoring System, respectively. Then, the participants fasted overnight, and their serum samples were collected the next day at the Healthy Examination Center of Fukang (International) Hospital in Tibet (Figure 1).

### 2.3 Physiological measurements

Before the physicians' measurements, all the participants (116 samples) were previously introduced to administer the questionnaire and then the physical examination. The serum samples were drawn after overnight fasting for complete blood count measurement. The routine blood indices included red blood cell count (RBC counts, 10∧12/L), absolute neutrophil count (NEUT#), neutrophil percentage (NEUT%), lymphocyte percentage (LYMPH%), absolute lymphocyte count (LYMPH#, 10∧9/L), and neutrophil/lymphocyte ratio (NLR).

### 2.4 ECG data acquisition and analysis

ECG recordings were obtained by a BioHarness Physiology Monitoring System (Zephyr BioHarness data acquisition system with 2-lead configuration, BIOPAC Systems, Inc., 42 Aero Camio, Goleta, CA, USA) with MP 150 hardware and sampled at 1 kHz and AcqKnowledge 4.2.1 software. Two electrodes were connected to the chest and the second left rib. The grounding electrode GND was connected to the right abdomen after the skin was disinfected with alcohol. The ECG was zeroed online, with a 50 Hz main notch, 0.5 Hz high-pass filtering, and 35 Hz low-pass filtering. Raw ECG was filtered using a bandpass filter (2 to 40 Hz). HRV analysis of the RR tachogram was performed for frequency domain (by power spectral analysis using fast Fourier transformation) and time domain measures using the Kubios (version 4.2).

### 2.5 Attention network test

The ANT task was based on the version employed by Fan et al. (2007), which can effectively measure the efficiency of all three attentional network effects (i.e., alerting, orienting, and executive) by combining a cued reaction time task (Posner, 1980) with a flanker task (Eriksen and Eriksen, 1974). Notably, the ANT shows moderate to high reliability (Fan et al., 2002), including in adults exposed to high-altitude hypoxia (Zhang et al., 2018b, 2021).

**Figure 2:**
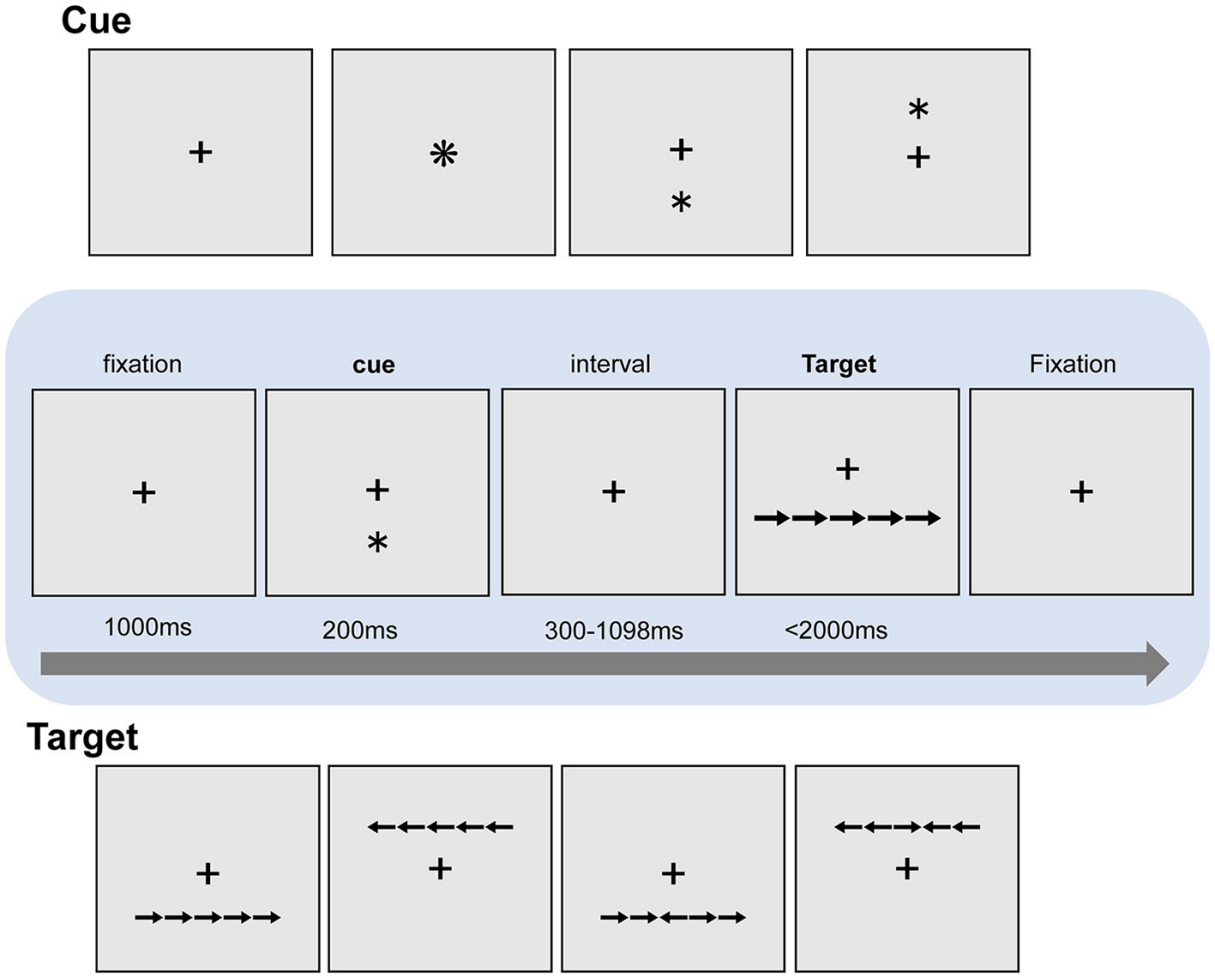
The ANT paradigm. A trial begins with a 1000 ms fixation period, and then a warning cue is presented for 200 ms. There is a short fixation period for a randomly variable duration after the warning cue, and then the target and flankers appear simultaneously. The target and flankers were presented until the participant responds, but for no longer than 2000 ms.

Participants performed one practice block of 10 trials followed by 6 experimental blocks, and each block consisted of 108 trials. The participants saw a “target” row of 5 horizontally aligned images of arrows and had to indicate the direction of the central arrow, which faced either the same or the opposite direction as the four surrounding “flanker” arrows (Figure 2). The target rows appeared either below or above the center of the screen. In each trial, the target row was preceded by one of three different cue types: (1) no-cue; (2) an asterisk in the center of the screen (central-cue); (3) an asterisk either above or below the center of the screen (spatial-cue), which was always consistent with the spatial location of the subsequent target row. Each trial began with a 1000 ms fixation, and then a warning cue was presented for 200 ms. There was a short fixation period for a randomly variable duration (300 ms−1098 ms) after the cue, and then the target and flankers appeared simultaneously. The target and flankers were presented until the participant responded, but for no longer than 2000 ms. After participants made a response, the target and flankers disappeared immediately. After a 1000 ms interval, the next trial began. The fixation cross remained at the center of the screen during the whole trial (The experimental flow chart is shown in Figure 2).

The efficiency of each attention network was estimated by comparing the response times (RTs) to the target stimulus under different conditions (different types of cues and different types of flankers). Note that in these tasks, accuracy is usually near the ceiling, so analysis is not usually performed (Veríssimo et al., 2022). Specifically, the alerting network efficiency was measured as a center-cue relative to the benefits of the trial with no-cue (RT speed-up), the efficiency of the orienting network was measured for spatial-clues relative to the center-cues (RT speed-up), and the benefits efficiency of the executive network was measured for incongruent flanks relative to the congruent flankers (the cost of RT deceleration); that is, the greater the interference caused by the flankers pointing in the opposite direction, the lower the efficiency of the executive network.

### 2.6 EEG recording and preprocessing

Continuous EEG was recorded at a sampling frequency of 1000 Hz by an ANT Neuro 64-electrode system (https://www.ant-neuro.com). Electrodes were positioned using the standard 10-20 system, and all impedances were kept below 5kΩ. The online reference electrode was placed on CPz, and the ground electrode was placed on FCz. Data processing and analyses were performed using the MATLAB toolbox EEGLAB (Delorme and Makeig, 2004) and Fieldtrip (Oostenveld et al., 2011) in MATLAB (version 2013b, The MathWorks). Raw data were downsampled to one-fourth (256 Hz) of the original sample rate before processing. Continuous EEG data were rereferenced offline to the average of all scalp electrodes, high-pass filtered at 0.1 Hz, and low-pass filtered below 40 Hz using a Basic FIR filter. For stimulus-locked analyses, the critical epochs ranged from −500 ms to 1000 ms relative to the onset of the stimulus, with −500 ms to 0 ms serving as the baseline. To remove ocular and muscle artifacts (including eye movements, blinks, heartbeat, and muscle artifacts), independent component analysis (ICA) was performed using the EEGLAB toolbox (on average, five components per participant were removed, SD = 2.0). Any remaining trials in which the amplitude exceeded ±100 μV were rejected. ERPs were computed by taking the average of all baseline-corrected trials (200 ms relative to stimulus onset) for each condition combined across the six experimental blocks. Amplitudes of P300 waves under target conditions were assessed by averaging single-trial amplitude values across time windows.

Time-frequency representations of the cleaned and epoched data were estimated per-trial using a 0.5 s Hanning taper applied in steps of 20 ms from −0.3 s to 0.8 s relative to stimulus onset (implemented in the Fieldtrip Toolbox, ft_freqanalysis function with the mtmconvol method). Frequencies ranged between 2 Hz and 40 Hz with a resolution of 2 Hz. We measured the absolute difference in oscillatory power in the alpha and theta bands among the no-cue, spatial-cue and center-cue conditions because the ANT test provided a very limited baseline period, i.e., the pre-cue interval (Balter et al., 2019).

For the “alerting” and “orienting” efficiency, we focused on alpha power (averaged across 8 Hz−14 Hz) and theta power (averaged across 4 Hz−8 Hz) in the interval between 100 ms post-cue onset to 300ms for center cues minus no cues (alerting) and spatial cues minus center cues (orienting). The regions of interest (ROIs) included the posterior channels for P3, P4, and Pz and the occipital channels for O1, O2, and Oz. For the “executive” efficiency, we examined the post-target differences in theta power (averaged across 4 Hz−8 Hz) between incongruent target flankers and congruent target flankers in the interval 300 ms−700 ms post-target onset. The ROIs included the frontal central channels for Fz, FCz, and Cz.

We computed the intertrial phase coherence from the Fourier representation using the following formula:


$$\begin{array}{l} ITC\left(f\right)=\frac{1}{n}\overset{n}{\underset{i=1}{\sum }}\frac{Z_{i}\left(f\right)}{{|Z}_{i}\left(f\right)|} \end{array}$$

Phase coherence for the no-cue condition was subtracted from each center-cue condition, the center-cue was subtracted from each spatial-cue condition, and the congruent criteria were subtracted from the incongruent condition. The resulting difference was utilized in the statistical analysis as the phase coherence of alerting, orienting, and executive, respectively.

ITC measured the temporal consistency of the phase value for a given frequency band at a certain time point. Phase coherence varies from 0 to 1, where 0 indicated absence of any EEG phase consistency across trials, and 1 indicated identical EEG phase consistency across trials (Delorme and Makeig, 2004).

### 2.7 Cognitive data calculation and analysis

Trials with incorrect responses and RTs that were not within the scope (mean ± 3 SD) were excluded from each participant. The network score was calculated according to the three operational definitions of network effects.

#### 2.7.1 Methods of behavior calculation

The efficiency of three attentional network scores based on the RTs was calculated as follows:

Alerting effect = RT_no − cue_ - RT_center − cue_

Orienting effect = RT_center − cue_ - RT_spatial − cue_

Conflict effect = RT_incongruent_ - RT_congruent_.

#### 2.7.2 Methods of EEG calculation

The contrasts for the alerting and orienting effects were reversed in the time domain, frequency domain, and time-frequency properties (i.e., alerting effect = center-cue - no-cue, orienting effect =spatial-cue - center-cue), and the contrasts for the executive control effect were the same as those for behavior (i.e., executive control effect = incongruent - congruent) (Fan et al., 2007).

### 2.8 Mediation analysis

Path modeling is a more flexible and powerful extension to the regression model where directional hypotheses about linear relationships between independent variables (RBCs) and dependent variables can be tested (three attention networks). It should be noted that path modeling does not provide evidence for the causality of such relationships. However, it may indicate whether the causal model under investigation is compatible with the data. The analyses were performed using online SPSS analysis software -SPSSAU.

All hypothesis tests conducted were two-tailed. We reported *X*^2^ fit statistics, RMSEA with its 90% CI and SRMR. RMSEA of < 0.05 and an SRMR below 0.1 imply a good fit. We also reported the CFI and the TLI, where values of CFI and TLI over 0.95 represented good fit. For model comparisons, we reported the robust (scaled) Satorra-Bentler *X*^2^ difference test. We also reported the BIC, which is penalized for the number of freely estimated parameters, favoring the least complex model. As a rule of thumb, a BIC difference over 10 is considered very strong evidence against the model with the highest BIC, 6 to 10 are considered strong evidence, 2 to 6 are considered positive evidence, and 0 to 2 are considered negligible evidence.

## 3 Results

### 3.1 Neurophysiological effect of hypoxic exposure on attention

**Figure 3:**
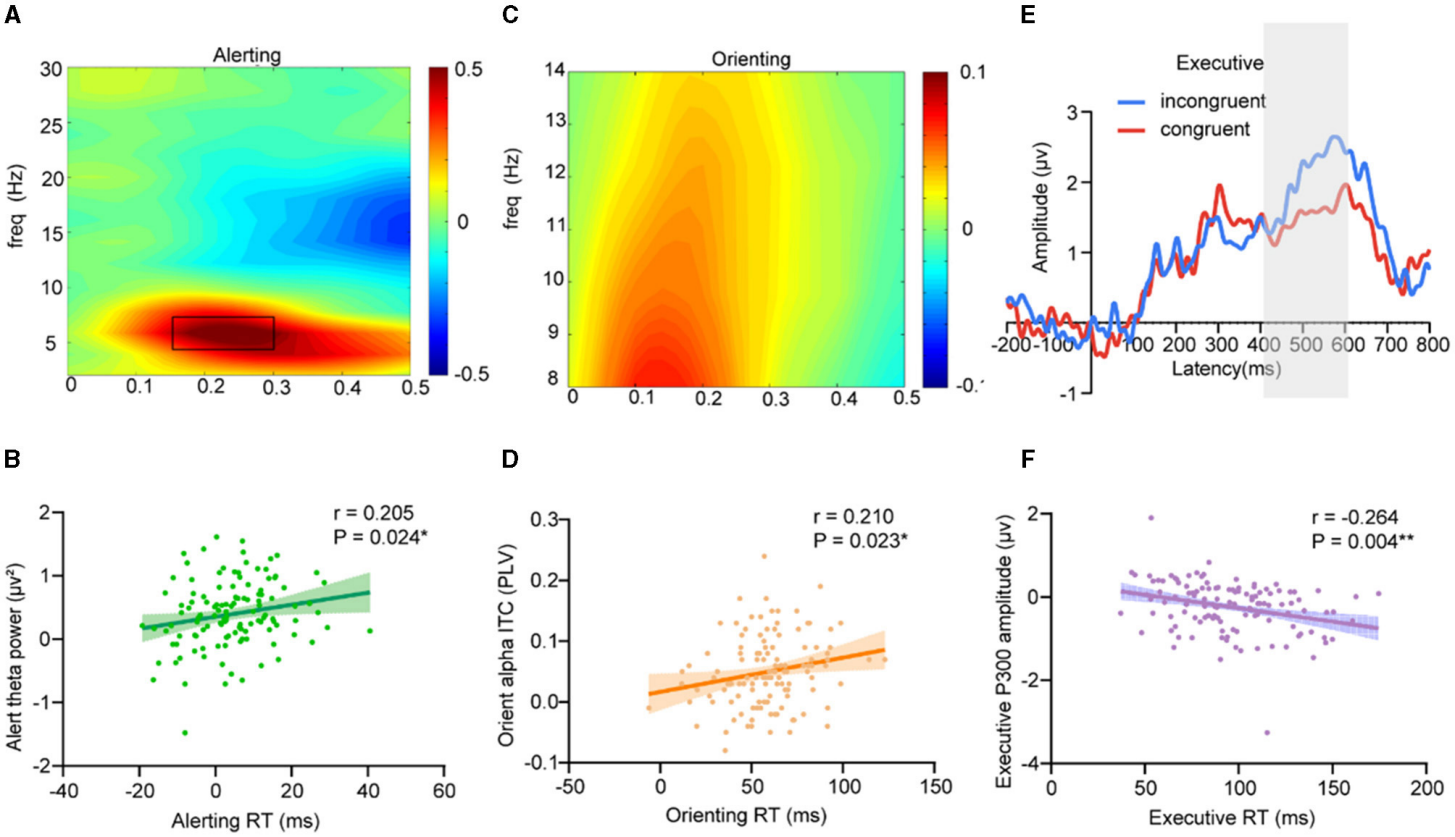
Neural markers of attention network performance. **(A, B)** Alerting efficiency showed a significantly positive correlation to the parietal and occipital alpha band (8 Hz−14 Hz) power in the interval 150 ms−300 ms post-cue onset for center cues vs. no cues (Pearson's correlation: *r* = 0.205, *P* = 0.024); **(C, D)** Orienting efficiency showed a significantly positive correlation to orienting alpha ITC (Parietal and occipital alpha (8 Hz−14 Hz) inter-trial coherence in the interval 150 ms−250 ms post-cue onset for spatial cues vs. center cues) (Pearson's correlation: *r* = 0.210, *P* = 0.023); **(E, F)** Executive efficiency showed a significantly negative correlation to P300 amplitude (400 ms−600 ms) (Pearson's correlation: *r* = −0.264, *P* = 0.004).

To examine the neurophysiological effect of hypoxic exposure on attention, we first sought to identify, a priori, key neural signatures that underlie the attention network in adults with hypoxic exposure. The results showed that the alerting efficiency was positively correlated with the parietal and occipital theta band (4 Hz−8 Hz) power (Figure 3A) in the interval 150 ms−300 ms post-cue onset for center cues vs. no cues (Pearson's correlation: *r* = 0.205, *P* = 0.024) (Figure 3B); orienting efficiency showed a significantly positive correlation to orienting alpha ITC (Parietal and occipital alpha (8 Hz−14 Hz) (Figure 3C) intertrial coherence in the interval 150 ms−250 ms post-cue onset for spatial cues vs. center cues) (Pearson's correlation: *r* = 0.210, *P* = 0.023) (Figure 3D); and executive efficiency showed a significantly negative correlation to P300 amplitude (Figure 3E) (Pearson's correlation: *r* = −0.264, *P* = 0.004) (Figure 3F).

### 3.2 Relationships between neurophysiological indicators and behavioral performance

**Figure 4:**
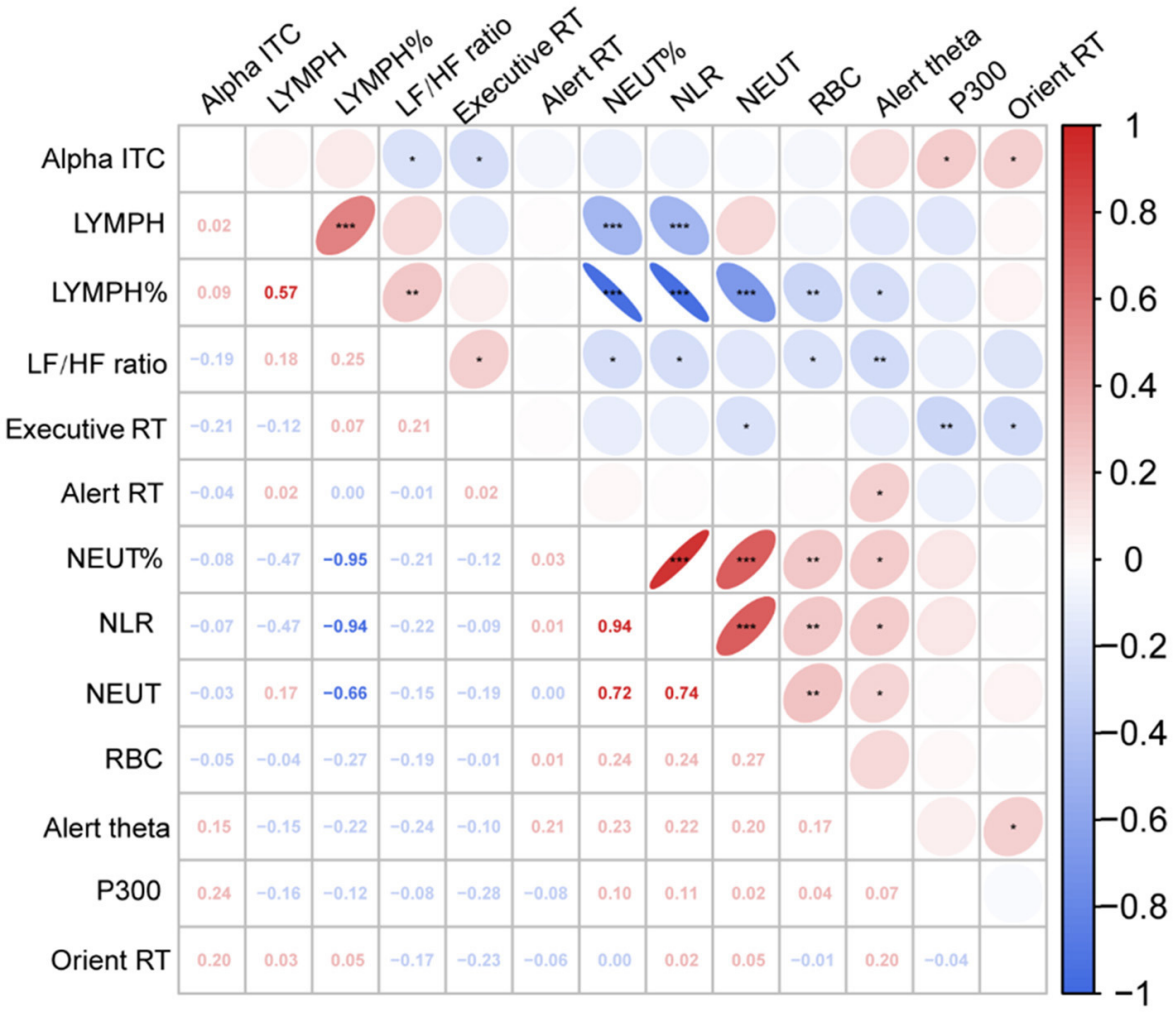
Partial correlation heat map among study variables. Executive RT, Executive response time; Alert RT, Alert response time; Orien RT, Orient response time; Alpha ITC, Orient alpha inter trail phase coherence; LF/HF Ratio, low frequency/high frequency ratio; RBC, red blood cell count; NLR, neutrophil-to-lymphocyte ratio; NEUT, neutrophilic granulocyte count; NEUT%, neutrophilic granulocyte percentage; LYMPH, lymphocyte count; LYMPH%, lymphocyte percentage.*P < 0.05; **P < 0.01; ***P < 0.001.

We calculated the partial Pearson's correlation coefficient to estimate the relationship between neurophysiological indicators and behavioral performance, controlled BMI, age, sex, and education (Figure 4). The results showed that RBCs was significantly positively correlated with NLR (*r* = 0.244, *P* =0.010), NLR was significantly negatively correlated with LF/HF (*r* = −0.215, *P* =0.023), and LF/HF was significantly correlated with executive RT (*r* = 0.214, *P* =0.024), Alert theta power (*r* = −0.239, *p* = 0.011) and Orient alpha ITC (*r* = −0.193, *P* = 0.042).

### 3.3 Mediation pathway model between RBCs and the attention network

**Figure 5:**
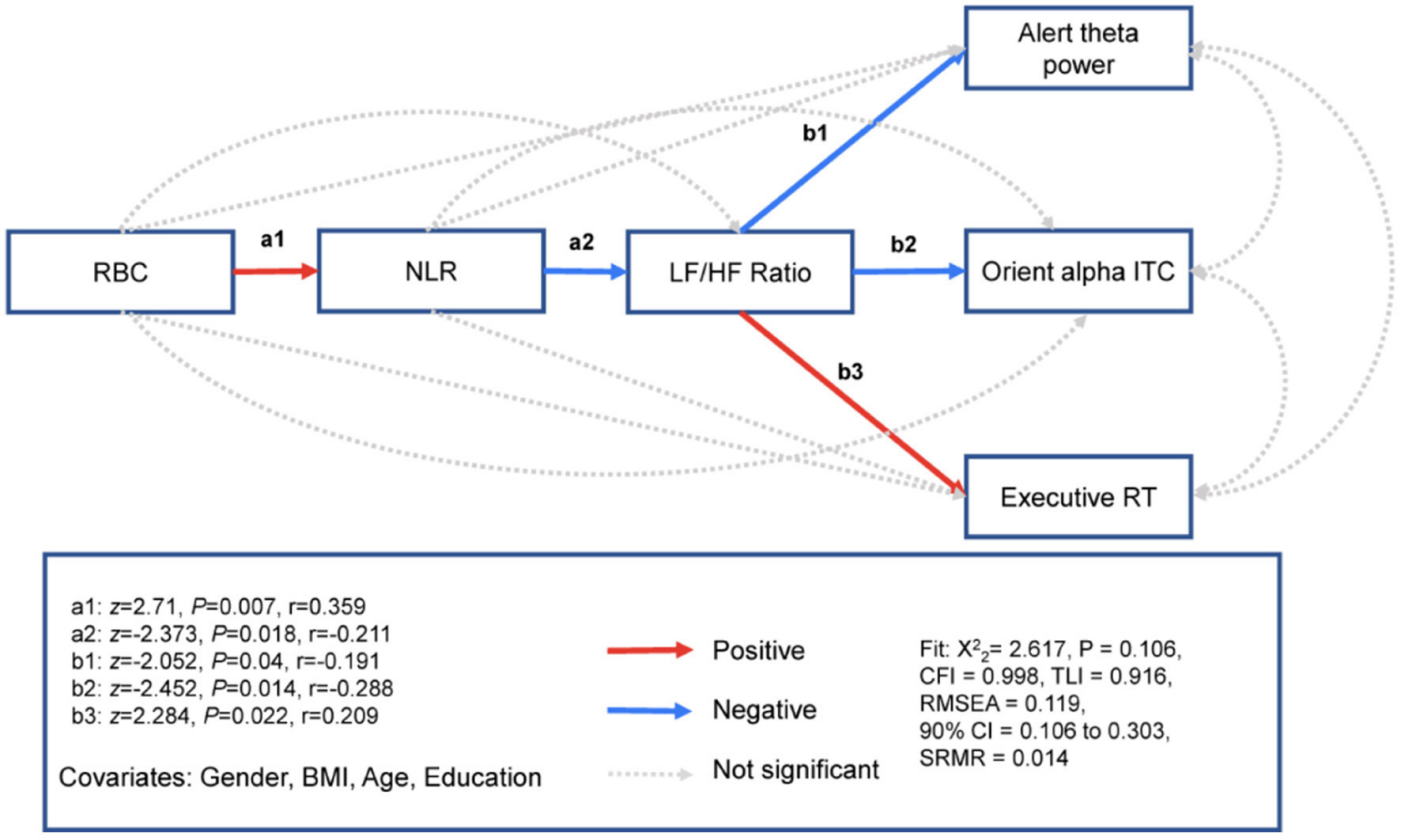
RBC associated with three attention networks, respectively. Path model showing that RBC specificity is associated with the neurophysiological markers of alerting, parietooccipital theta power; the neurophysiological markers of orienting, parietooccipital alpha ITC; and executive RT through inflammation and HRV. z, standardized path coefficient; r, Pearson's r effect size. *N* = 116.

To examine the effect of oxygen transport capacity (i.e., RBC count) on the attention network, we used path modeling to examine whether RBC count was related to three attention networks through the inflammatory markers NLR and HRV. The mediation pathway analyses showed that RBC count was related to higher NLR (Effect = 0.399, S.E. = 0.147, *z* = 2.71, Pearson's effect size *r* = 0.359, *P* = 0.007) but not the LF/HF ratio (Effect = 0.094, S.E. = 0.247, *z* = 0.379, *r* = 0.035, *P* = 0.704) or the three attention networks for alerting (Effect = 0.135, S.E. = 0.127, *z* = 1.064, *r* = 0.143, *P* = 0.287), orienting (Effect = −0.01, S.E. = 0.014, *z* = −0.736, *r* = −0.099, *P* = 0.462) or executive control (Effect = 0.003, S.E. = 0.007, *z* = 0.505, *r* = 0.067, *P* = 0.614). The NLR was related to the LF/HF ratio (Effect = −0.504, S.E. = 0.213, *z* = −2.373, *r* = −0.211, *P* = 0.018) but not related to the three attention networks for alerting (Effect = 0.137, S.E. = 0.079, *z* = 1.728, *r* = 0.162, *P* = 0.084), orienting (Effect = −0.009, S.E. = 0.009, *z* =−1.027, *r* = −0.096, *P* = 0.305), or executive control (Effect = −0.002, S.E. = 0.004, z =-0.539, *r* = −0.05, *P* = 0.59). The LF/HF ratio was related to three attention networks for alerting (Effect = −0.067, S.E. = 0.033, *z* =−2.052, *r* = −0.191, *P*= 0.04), orienting (Effect = −0.009, S.E. = 0.004, *z* = −2.452, *r* = −0.228, *P* = 0.014), and executive control (Effect = 0.004, S.E. = 0.002, *z* = 2.284, *r* = 0.209, *P* = 0.022). The model fit was excellent (Figure 5 and Table 2).

**Table 2:** Inflammation, HRV, and demographic variables as predictors for the effect of RBCs on three attention networks.

**Outcome**	**Predictor**	**Effect**	**s.e**.	**z**	***P* (>|z|)**	**r**
Alert theta power	RBC	0.135	0.127	1.064	0.287	0.143
	NLR	0.137	0.079	1.728	0.084	0.162
	LF/HF Ratio	−0.067	0.033	−2.052	**0.04**	**−0.191**
	Gender	0.061	0.138	0.441	0.659	0.058
	Age	−0.006	0.014	−0.407	0.684	−0.037
	BMI	−0.005	0.015	−0.327	0.744	−0.031
	Education	0.067	0.052	1.289	0.198	0.12
Orient alpha ITC	RBC	−0.01	0.014	−0.736	0.462	−0.099
	NLR	−0.009	0.009	−1.027	0.305	−0.096
	LF/HF Ratio	−0.009	0.004	−2.452	**0.014**	**−0.228**
	Gender	0.008	0.015	0.505	0.613	0.066
	Age	−0.001	0.002	−0.328	0.743	−0.03
	BMI	0.001	0.002	0.331	0.74	0.032
	Education	−0.006	0.006	−1.072	0.284	−0.1
Executive RT	RBC	0.003	0.007	0.505	0.614	0.067
	NLR	−0.002	0.004	−0.539	0.59	−0.05
	LF/HF Ratio	0.004	0.002	2.284	**0.022**	**0.209**
	Gender	−0.002	0.007	−0.206	0.837	−0.026
	Age	0.001	0.001	1.583	0.113	0.144
	BMI	0.002	0.001	1.961	**0.05**	**0.184**
	Education	−0.002	0.003	−0.72	0.471	−0.066
NLR	RBC	0.399	0.147	2.71	**0.007**	**0.359**
	Gender	0.339	0.161	2.111	**0.035**	**0.271**
	Age	0.018	0.017	1.056	0.291	0.098
	BMI	−0.006	0.018	−0.343	0.731	−0.033
	Education	−0.056	0.062	−0.904	0.366	−0.085
LF/HF Ratio	NLR	−0.504	0.213	−2.373	**0.018**	**−0.211**
	Gender	−0.651	0.271	−2.407	**0.016**	**−0.217**
	Age	−0.025	0.04	−0.639	0.523	−0.059
	BMI	0.003	0.04	0.071	0.943	0.007
	Education	−0.035	0.146	−0.24	0.81	−0.022
Orient alpha ITC	Alert theta power	0.004	0.003	1.458	0.145	0.123
Alert theta power	Executive RT	−0.001	0.001	−0.586	0.558	−0.048
Executive RT	Orient alpha ITC	−0.000	0.000	−1.949	0.051	−0.164

N = 116. Significant paths are emboldened. Robust model fit indices: X^2^ = 2.617, P = 0.106, CFI = 0.998, TLI = 0.916, RMSEA = 0.119, 90% CI = 0.106 to 0.303, SRMR = 0.014. BMI, body mass index; Orienting alpha ITC, orienting alpha inter trail phase coherence; LF/HF Ratio, low frequency/high frequency ratio; RBC, red blood cell count; NLR, neutrophil-to-lymphocyte ratio; Effect, unstandardized path coefficient; s.e., robust standard error; z, standardized path coefficient; r, Pearson's r effect size; 95% CI, 95% confidence interval of the effect size.

We report chi-square (*X*^2^) fit statistics, the root mean squared error of approximation (RMSEA), standardized root mean square residual (SRMR), the comparative fit index (CFI), the Tucker–Lewis index (TLI), and the Bayesian information criterion (BIC) (see “Materials and Methods”). Robust fit statistics indicated a good fit for the model with both predictors ($X_{2}^{2}$ = 2.617, *P* = 0.106, CFI = 0.998, TLI = 0.916, RMSEA = 0.119, 90% CI = 0.106 to 0.303, SRMR = 0.014).

## 4 Discussion

In this study, ANT was used in combination with EEG, ECG, and blood routine indicators to explore the relationship between hypoxic-induced neurophysiological response and attentional function of Tibetan immigrants. We first identify, a priori, key neural markers that underlie the attention network in Tibetan immigrants. Furthermore, we explored the effect of oxygen carrying on attention networks, and pathway analysis showed that the RBCs as oxygen carrying cells affected the three attention networks through inflammation and HRV. All these observations provide experimental evidence for the existence of a neuroimmune pathway rooted in RBCs, which represents oxygen transport capacity and is the basic physiological mechanism that determining human attention performance in a high-altitude environment.

RBCs, as oxygen-carrying cells, indirectly affected the three attention networks. Inflammation and HRV mediate this relationship. Given that HRV and inflammatory markers are important indicators of center-peripheral integration and homeostasis (Taylor et al., 2010), we believe that the key to the influence of RBCs on the attention network of immigrants may be to improve the oxygen transport capacity and maintain homeostasis by increasing RBCs within the normal range. Blood consists of 80% RBCs, especially hemoglobin, which carries oxygen to maintain brain functions. RBCs also play an important role in inflammation and the immune system (Lam et al., 2021; Xu et al., 2022). Our previous study also found that RBCs not only triggered internal-sensory representation of the insula association of orienting but also induced immune responses corresponding to executive control (Xue et al., 2022). Interoceptive inference (that is, the approximate Bayesian inference about the state of the internal) can be used to explain how organisms regulate homeostasis in hypoxic environments by making decisions about external perception and proprioception, in which the insular cortex plays a key role (Seth, 2013; Gu and FitzGerald, 2014). In a hypoxic environment, the brain senses hypoxia externally, increasing the oxygen-carrying capacity of RBCs as compensation (Wood and Johansen, 1972; Raberin et al., 2022; Villafuerte et al., 2022), and then the internal environment of the body is homogenized and controlled by autonomic reaction. When the brain perceives the gap between external perception and proprioception, it makes decisions through interoceptive inference, which is the basis of behavioral response (Gu and FitzGerald, 2014).

HRV may be the key to regulating the relationship between oxygen transport capacity and attention networks. The response of the autonomic nervous system (ANS) is the key to acclimation during high-altitude exposure, which were often evaluated by HRV (Zhang et al., 2014; Temme et al., 2023). High-altitude exposure is an effective activator of the ANS (Roche et al., 2002; Taralov et al., 2018; Temme et al., 2023), resulting in decreased parasympathetic tone and increased sympathetic tone, and decreased HRV. HRV as one of the robust resilience markers (Walker et al., 2017) indicates the activity of the vagus nerve (i.e., primary parasympathetic nerve), which reflects the influence of the parasympathetic nervous system (PNS) on cardiac regulation (Laborde et al., 2017). Higher HRV physiologically indicates lower stress, higher ability to cope with stress, appropriate adaptation and flexibility to environmental changes, and more effective self-regulation (Shaffer and Ginsberg, 2017; Christodoulou et al., 2020). HRV can also predict the outcome of cognitive tests representing executive function. The neurovisceral integration model (NIM) view suggests that the subfrontal inhibitory circuit is connected to the heart via the vagus nerve, which provides inhibitory input to the heart, and that the prefrontal cortex (PFC) is also associated with the inhibitory capacity of executive function (Thayer et al., 2009). Furthermore, based on consistent evidence from many studies, a decrease in cardiac parasympathetic activity has been reported in both rat and human studies because of high-altitude exposure (Siebenmann et al., 2017; Beltrán et al., 2020).

Furthermore, the complex combination of physiological, behavioral, emotional, and cognitive processes involved in self-regulation and adaptation may have a common basis in that HRV will be linked to all these various forms of regulation (Holzman and Bridgett, 2017). HRV plays a key role in homeostasis, and there is evidence that the parasympathetic branch of the autonomic nervous system (ANS) plays a particularly critical role in modulating inflammation (Borovikova et al., 2000). Specifically, the parasympathetic nervous system (PNS) is involved in monitoring current levels of inflammation and reducing the production of inflammatory cytokines through rapid afferent and efferent signals from the vagus nerve (Tracey, 2002). LF/HF ratio is a sensitive index of HRV at high-altitude (Zhang et al., 2014), is an indicator of sympathetic vagus nerve balance or a reflection of sympathetic nerve regulation (Heathers, 2014). Elevated LF/HF ratios may indicate ANS dysfunction, leading to sympathetic vagus imbalance and sympathetic overweight (Kuriyama et al., 2010; Chen et al., 2011). HRV reflects the regulatory function of ANS and the brain's control over behavior and peripheral physiology and is a good biomarker of individual adaptation (Zhang et al., 2014). The degree of inflammatory response is important because an extreme or inadequate response can cause varying degrees of harm to an individual (Williams et al., 2019). In our results, the overall NLR range was < 3. Here, it should be carefully explained that the increase of RBCs is accompanied by the increase of NLR. Some studies have shown that NLR < 3 belongs to the normal range (Bahrami et al., 2019).

We also observed the effects of demographic variables on the attention network and immune system of immigrants. Specifically, we observed that gender was significant in the prediction of LF/HF ratios and NLR, while BMI also showed significance in the prediction of Executive RT. The effect of gender may reflect biological differences in neuropsychological functioning and immune system regulation. Previous studies have suggested that gender may play a key role in the interactions between the autonomic nervous system and the immune system (Ferguson et al., 2013; Fransen et al., 2017), which in turn affects the LF/HF ratio and the NLR. Further studies could explore the underlying mechanisms of these gender differences to better understand the effects of these variables on attentional networks and immune indexes. And the significance of BMI in the prediction of Executive RT may reflect the complex relationship between BMI and executive function. Several studies have suggested that changes in BMI may be associated with adjustments in executive function (Stanek et al., 2013; Mac Giollabhui et al., 2020), and our findings emphasize the importance of this association. Overall, the significant effects of these demographic variables provide the impetus for further research to delve into the physiological basis of the relationship between gender and BMI and the attentional network and immune system. It also highlights the importance of the need to consider these factors in more detail in future studies to better understand the role of individual differences in the neuroimmune regulation of immigrants.

Our results confirm the theory of the neurovisceral integration model (NIM) in high-altitude. According to Thayer and Lane (2000) on neuro-visceral integration (Thayer et al., 2009; Park and Thayer, 2014), the strong vagal regulation of the heart is related to the effective function of self-regulating neural circuits, which enable the body to respond quickly and flexibly to various environmental demands. Our results suggest that HRV (LF/HF ratio) can predict performance of executive control behavior, as well as brain activity during alerting and orienting. It is worth noting that the theta power represents the cognitive resources related to attention (Thayer and Lane, 2000; Thayer et al., 2009; Park and Thayer, 2014), alpha ITC represents the selective inhibition of target information in sensory input (inhibition of irrelevant information and discrete cognitive processes) (Hanslmayr et al., 2005; Busch et al., 2009; Gutteling et al., 2022), P3 may be a neurophysiological marker sensitive to high-altitude exposure (Wesensten et al., 1993; Singh et al., 2004; Wang et al., 2021). P3 amplitude reflects the allocation of attentional resources (Polich, 2007; Fogarty et al., 2018), and a decrease in amplitude indicates a reduction in the remaining effective indicator of mental resources (Magliero et al., 1984; Clayson and Larson, 2011; Schmidt-Kassow et al., 2013; Fogarty et al., 2018). These also indicate that immigrants with better oxygen transport ability have more cognitive resources in attention activities and better selective inhibition of target information, which is realized through the regulation of the autonomic nervous system on the homeostasis of the organism. Previous studies also found that cardiac vagus nerve (CVN) activity is associated with early attention orienting and promotes flexible absorption of effective orientation information (Sørensen et al., 2019). These results suggest that RBCs in high-altitude immigrants is associated with better cardiac vagal tone, which is related to the effective function of attentional self-regulating neural circuits, increasing cognitive resources in the process of selective attention, and improving the selective attention ability to target information.

Limitations of this study and several problems need to be considered in the future work. First, the results of this study need to be further verified and extended, for example (1) to test other aspects of cognitive function; (2) Study individual differences in cognitive function caused by exposure; Secondly, the present study only identified a neuroimmune pathway of RBCs affecting attention function in high-altitude immigrants, and we do not know whether this pathway is also present in low-altitude populations or in age- and gender-matched indigenous Tibetan residents, and future studies could give the results of differences in the study variables between the indigenous Tibetan residents and immigrant populations and enrich the results of the possible adaptive differences. (3) We did not consider the factors that influence HRV, such as smoking, alcohol consumption, and exercise. These may have affected our overall understanding of how HRV changes under different lifestyle conditions and environmental influences. Therefore, future studies should consider these factors in more detail and explore their relationship with HRV to provide a more comprehensive understanding of the mechanisms of HRV variation in high-altitude immigrants. However, these do not affect the goal of the whole study, which is to seek intervention measures for high-altitude migration. And, in the future, we can also seek personalized interventions from the perspective of individual differences. The mechanism revealed in this study may provide reference for future interventions in high-altitude immigrants from the point of view of erythrocyte function, for example, by increasing the RBCs in high-altitude immigrants within the normal range through dietary therapy or other interventions.

## 5 Conclusion

In summary, we have identified a neuroimmune pathway rooted in erythrocytes, representing oxygen transport capacity as the basic physiological mechanism that determines human attention performance at high-altitude. Inflammation and HRV mediate the relationship between erythrocytes and attentional function. Our results also confirm the theory of neurovisceral integration model (NIM) at high-altitude. Overall, these findings provide valuable insights into solving the health problems of immigrants who are chronically exposed to high-altitude environments and provide a scientific basis for further research in this area.

## Data availability statement

The raw data supporting the conclusions of this article will be made available by the authors, without undue reservation.

## Ethics statement

The studies involving humans were approved by the Ethics Committee of Tibet University. The studies were conducted in accordance with the local legislation and institutional requirements. The participants provided their written informed consent to participate in this study.

## Author contributions

N-NW: Formal analysis, Investigation, Methodology, Resources, Software, Visualization, Writing – original draft, Writing – review & editing. S-FY: Methodology, Writing – original draft. PD: Methodology, Writing – review & editing. RS: Investigation, Writing – review & editing. HL: Investigation, Writing – review & editing. H-LM: Methodology, Writing – review & editing, Conceptualization, Project administration. ML: Conceptualization, Writing – review & editing, Supervision. D-LZ: Conceptualization, Supervision, Writing – review & editing, Project administration.
